# Herpes Zoster Burden in Canadian Provinces: A Narrative Review and Comparison with Quebec Provincial Data

**DOI:** 10.1155/2018/3285327

**Published:** 2018-10-21

**Authors:** Marie-Claude Letellier, Rachid Amini, Vladimir Gilca, Gisele Trudeau, Chantal Sauvageau

**Affiliations:** ^1^Université Laval, Quebec City, Canada; ^2^Institut National de Santé Publique du Québec, Quebec City, Canada; ^3^Centre Hospitalier Universitaire de Québec—Université Laval, Quebec City, Canada

## Abstract

**Background:**

The main aim of this review was to assess incidence rates and trends of medically attended and death cases of herpes zoster in Canada.

**Methods:**

The search was conducted in five databases (PubMed, Cochrane, Embase, PsycNET, and Web of Science). Data on herpes zoster-related consultations and hospitalisations and deaths were also extracted from three Quebec provincial administrative databases (RAMQ, MED-ECHO, and ISQ).

**Results:**

The electronic search yielded 587 publications. Seventeen publications satisfied inclusion criteria. These publications reported data from eleven studies. Ten studies used provincial databases, and one study used the Canadian Primary Care Sentinel Surveillance Network electronic database. Seven studies evaluated overall rates of medically attended cases (consultations and hospitalisations). Four of these studies reported an increase in rates of medically attended cases during the study period; one study reported stable rates, and two studies reported only an average rate. The rates varied from 316 to 450/100,000 p.y. The Quebec analysis shows similar rates with a slight decreasing trend (from 369 to 350/100,000 p.y.). Incidence rates of consultations were reported separately in three studies. Two studies reported an increase in rates (from 258 to 348/100,000 p.y. and from 324 to 366/100,000 p.y.), and the third study reported a decrease (from 525 to 479/100,000 p.y.). Hospitalization rates were reported separately in two studies, both reporting a decrease (from 12 to 8 cases/100,000 p.y. and from 9 to 4 cases/100,000 p.y.). Quebec data also showed a decrease, from 9 to 6 cases/100,000 p.y. One study reported herpes zoster-related deaths. In this study, the reported death rate was 0.7/1,000,000 p.y. in the overall population and 5.5/1,000,000 p.y. in those aged ≥65 years. Quebec analysis showed a death rate of 1.2/1,000,000 p.y. in the overall population and 8.6/1,000,000 p.y. in those aged ≥65 years.

**Conclusions:**

The results of the reviewed studies and our analysis of Quebec provincial data indicate important variations in the reported overall incidence rates of medically attended herpes zoster cases in Canada. The trends in time are heterogeneous in studies in which hospitalisations and medical consultations were pooled together. We observed a decrease in hospitalization rates and a slight increase in consultation rates in studies reporting hospitalisations and consultations separately. These results consolidate the understanding of the herpes zoster burden in Canada and might be used as a tool in decision-making regarding future preventive interventions.

## 1. Background

Varicella zoster virus results in varicella (chickenpox) as primary infection. The virus remains latent in the sensory ganglia and can reactivate later in life, causing herpes zoster, also known as shingles. While children are the main contributors to varicella burden, the elderly are particularly affected by herpes zoster. The implementation of the varicella vaccine in childhood vaccination programs has substantially decreased disease incidence and related complications, including hospitalizations and deaths [1]. The potential impact of this decrease on the herpes zoster burden is not well understood. Some authors have raised the concern that high varicella vaccine coverage is followed by a decrease of virus circulation, and as a result, a decrease of natural boosting could lead to an increase in herpes zoster incidence [2, 3]. Uncertainties regarding this hypothesis led to an increase in the number of studies on the herpes zoster epidemiology [4, 5].

Varicella vaccine has been available in Canada since 1999 and has been largely used in provincial publicly funded childhood vaccination programs since the early 2000s. In the province of Quebec, such a program was implemented in 2006. A few years after program implementation, a decrease in consultations, hospitalizations, and deaths associated with varicella was reported [6]. The first live attenuated herpes zoster vaccine was approved for use in Canada in 2009. This vaccine is recommended for adults aged 60 years and over and is authorized for use in some subpopulations at risk for herpes zoster aged 50 years and over. The use of the live attenuated vaccine is limited because of several reasons including relatively low short- and midterm efficacy [7], its relatively high price, the recommendation to an age group in which vaccines' uptake is relatively low, the cost-effectiveness of a publicly funded programs remains questionable, and it is not publicly funded in most Canadian provinces. By 2018, only one out of ten Canadian provinces implemented a publicly funded herpes zoster immunization program [8]. The recent approval of a new promising recombinant herpes zoster vaccine [9] raises the question about its optimal use and potential impact on herpes zoster burden.

We conducted a review of Canadian studies examining herpes zoster burden. The main aim of this review was to assess incidence rates and trends of medically attended and death cases related to herpes zoster in Canadian jurisdictions with no or very low herpes zoster vaccination coverage at the time of study conduction. Moreover, we extracted and analysed data on the same issue from the Quebec provincial administrative databases. It was thought that such a review and analysis will provide useful information for (i) a better understanding of the impact of varicella vaccination on the epidemiology of herpes zoster, (ii) an estimation of herpes zoster burden, (iii) future cost-effective estimations of different potential immunisation programs, and (iv) decision-making regarding herpes zoster prevention.

## 2. Methods

### 2.1. Literature Search, Selection, and Eligibility Criteria

The main search for this systematic review was conducted on May 24, 2018. Five databases (PubMed, Cochrane, Embase, PsycNET, and Web of Science) were searched, using the keywords “shingles or herpes zoster or zona” and “epidemiology or incidence or burden” and “Canada.” We also conducted several additional searches based on a review of citations from relevant publications. No language, publication year, or age group restrictions were applied. After conducting the first search, all titles were reviewed for relevance. After excluding nonpertinent titles (e.g., limited to diagnosis, clinical manifestation, treatment, or clinical trial reports), we read all abstracts, and for those meeting the outcomes of interest, full articles were read to determine their eligibility for inclusion. To be eligible, the articles should have presented Canadian data on herpes zoster-related consultations, hospitalizations, and/or deaths. Two authors (MCL and VG) reviewed selected articles for eligibility and resolved discordances by discussion.

### 2.2. Quebec Data

We extracted data on herpes zoster-related consultations and hospitalisations, hospitalisations separately, and deaths from three provincial administrative databases: RAMQ (Régie de l'Assurance Maladie du Québec), MED-ECHO (Maintenance et Exploitation des Données pour l'Étude de la Clientèle Hospitalière), and ISQ (Institut de la statistique du Québec). The data received from RAMQ were aggregated and did not allow us to separate consultations from hospitalisations. Data on consultations and hospitalisations (RAMQ database) and hospitalisations separately (MED-ECHO database) were available for the period from 1996 to 2015 and on death causes for the period from 1996 to 2013 (ISQ database). Herpes zoster diagnoses with the code 053.x in the ninth edition and B02.x in the tenth edition of the International Classification of Diseases (ICD) were extracted. For hospitalisations, only cases with herpes zoster as the principal or first secondary diagnosis were retained for this analysis. An incident case was defined as the first herpes zoster diagnosis during a calendar year. To verify for a potential double counting, a second analysis was performed by defining as incident only the first-time herpes zoster diagnosis during the entire study period (1996–2015). A death was included in the analysis if herpes zoster was the underlying cause of death.

Incidence rates of consultations and hospitalisations and deaths were calculated for the overall provincial population and for people aged 65 years and over. Overall incidence rates were standardized for age with the 2007 Quebec population as reference (data obtained from ISQ). Both Quebec analysis and reviewed studies were restricted exclusively to anonymous data. Thus, no research ethics board approval was required.

## 3. Results

**Figure 1:**
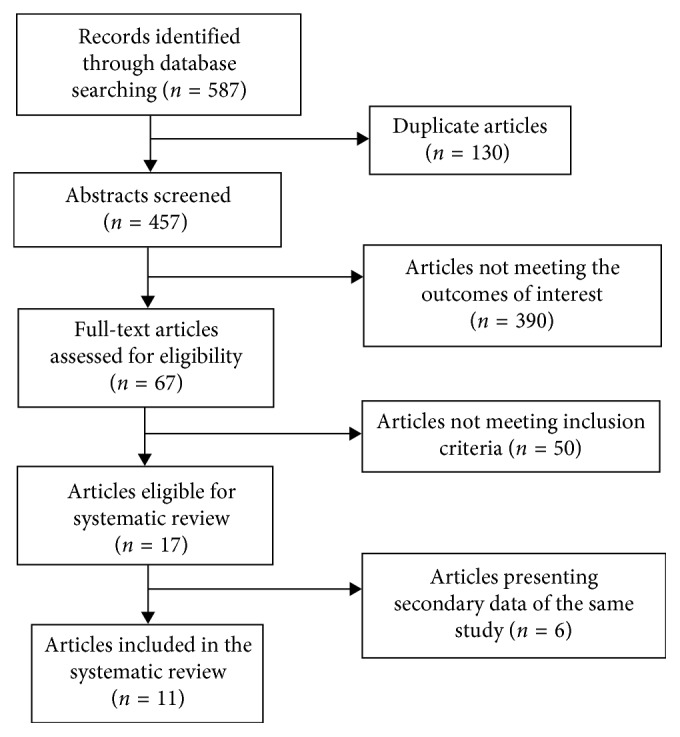
Flow chart of study selection for systematic review.

The electronic database search yielded 587 hits; 130 were duplicates in two or more databases and were excluded. The remaining 457 citations were screened by title and abstract, and 390 were excluded because of lack of any preestablished outcome or population of interest. The 67 articles potentially containing outcomes of interest were read fully. Seventeen of them satisfied inclusion criteria and were included in the review (Figure 1). These seventeen articles reported data from ten original studies that were conducted in 4 Canadian provinces (British Columbia [4, 10], Manitoba [11–13], Alberta [14–16], and Ontario [17, 18]), and one study was conducted based on the Canadian Primary Care Sentinel Surveillance Network electronic database (CPCSSN) [19]. The main features of the included studies are presented in Table 1.

**Figure 2:**
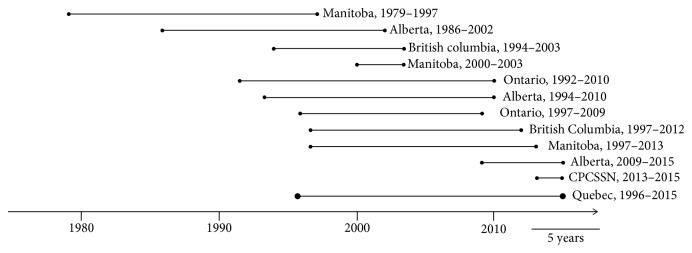
Studies' periods and duration.

There was wide variation between studies' periods (Figure 2). Some studies considered one unique period [4, 11–13, 15, 16, 18, 19], while others subdivided study periods into pre- and postvaricella vaccination program implementation [10, 14, 17]. Ten studies used provincial administrative databases and ICD classification (Table 1). However, there were some differences in the definition of an incident case in included studies. Some studies reported only the first episode [10, 11, 15, 17, 18], and others counted a subsequent episode if separated from the first one by at least 6 months [14], one year [4], or two years [13]. Studies reported crude and/or standardized incidence rates for overall population or rates by age groups. Incidence rates standardized for age were generally lower than crude ones. Finally, three different age criteria were used when assessing herpes zoster incidence rates among the elderly: 60 years and over [17], 65 years and over [4, 11, 12, 19], and 66 years and over [18].

**Figure 3:**
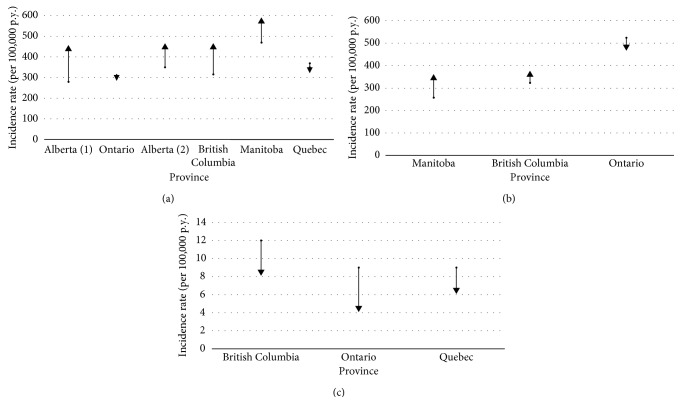
Incidence rates of hospitalisations and consultations (a), consultations (b), and hospitalisation (c) for herpes zoster by Canadian Province.

Seven studies evaluated incidence rates of medically attended cases (consultations and hospitalisations) [10, 13–18]. Four of these studies reported an increase in incidence rates of medically attended cases during the study period; one study reported relatively stable rates (Figure 3(a)), and two studies reported only the average incidence rate for the analysed period. In the five studies reporting the rates trend, the incidence rates of medically attended cases varied from 316 to 570 cases per 100,000 person-years (p.y.). One of these studies [13] limited eligibility criteria to cases aged 20 years and over. This study reported the highest incidence rates. When excluding this study, the incidence rates of medically attended cases varied from 316 to 450 cases per 100,000 p.y. The analysis of Quebec data shows similar incidence rates of medically attended cases with a slight decreasing trend (from 369 to 350 cases per 100,000 p.y.; rate ratio: 0.95 (95% CI: 0.94–0.96)). This trend is due to a slight decrease in incidence rates in individuals under the age of 60. The most pronounced decrease was observed in children aged 0–9 years in the postvaricella vaccination period (2006–2015) when compared to the prevaccination period (from 83 to 65 cases per 100,000 p.y.; rate ratio: 0.79 (95% CI: 0.76–0.82)).

Incidence rates of consultations were reported separately in three studies (Figure 3(b)). Two studies reported an increase in consultations rates (from 258 to 348 cases [11] and from 324 to 366 cases [4] per 100,000 p.y.), and the third study reported a decrease (from 525 to 479 cases per 100,000 p.y. [17]).

Hospitalization rates were reported separately in two studies, both reporting a decrease (from 12 to 8 cases per 100,000 p.y. [4] and from 9 to 4 cases per 100,000 p.y. [17]; Figure 3(c)). Quebec data also showed a decrease, from 9 to 6 cases per 100,000 p.y. Three other studies reported a decrease of the percentage of hospitalized cases from 4-5% to 2-3% [10, 14, 15] without presenting hospitalisation rates.

All studies reported higher incidence rates for consultations, hospitalisations, and overall consultations and hospitalisations in the older population (60–66 years and over) when compared to younger population (Table 1).

Only one study reported herpes zoster-related deaths. In this study, the reported death rate was 0.7 per 1,000,000 p.y. in the overall population and 5.5 per 1,000,000 p.y. in those aged 65 years and over [4]. Quebec analysis shows a death rate of 1.2 per 1,000,000 p.y. in the overall population and 8.6 per 1,000,000 p.y. in those aged 65 years and over.

The second analysis of Quebec data with a restrictive definition of an incident case as the first-time consultations and hospitalizations for herpes zoster during the entire study period brought relatively little change to the results of the primary analysis; the overall number of cases decreased by less than 9% (from 543,290 to 495,111 cases), without a change in the general trend.

One study evaluated the prevalence of herpes zoster in patients with and without common chronic diseases. The age- and sex-adjusted herpes zoster prevalence in patients with chronic diseases was statistically significantly higher when compared to those observed in individuals without chronic diseases. Namely, the prevalence ratio was 1.57 for patients with diabetes, 1.83 for those with chronic obstructive pulmonary disease, 2.58 in case of neoplasm, and 6.46 for those with HIV/AIDS [19].

A recent Canadian study evaluated the effectiveness of the live attenuated herpes zoster vaccine in individuals aged 50 years and older. In this study, the vaccine effectiveness decreased from year to year, and by the fifth year, the vaccine offered no protection against the incident disease. The adjusted vaccine effectiveness in years one to five following vaccination, respectively, was 50.0% (95% CI: 44.7, 54.8), 34.5% (95% CI: 27.3, 41.0), 31.5% (95% CI: 21.8, 39.9), 30.5% (95% CI: 13.9, 44.3), and 14.0% (95% CI: −21.0, 39.0) [16].

## 4. Discussion

We searched publications in peer-reviewed scientific papers available in the five most comprehensive databases. This search found 11 original Canadian studies conducted in four different provinces (British Columbia: 2 studies; Alberta: 3 studies; Manitoba: 3 studies; and Ontario: 2 studies), and one study used the Canadian Primary Care Sentinel Surveillance Network database. These studies include data from 1979 to 2015. Over time, in Canada, a trend of a decrease in hospitalization rates and a slight increase in consultation rates was observed. The shift toward ambulatory care during the last decades and the availability of antiviral treatments can explain the decrease in hospitalizations. Higher chances of survival of immunosuppressed patients and an aging population can explain at least partially the reported increase in herpes zoster consultations in some but not all studies. When medically attended cases were analysed and presented in an aggregated manner (consultations and hospitalisations), some discrepancies were observed among studies' results. Four studies reported an increase, one study reported no change, while Quebec analysis showed a slight decrease of the incidence of all medically attended cases. These discrepancies might be explained by different time periods analysed, by different definitions used for an incident case, and by the methodology used when analysing and presenting data (i.e., crude and/or standardized incidence for age).

The Canadian publications available suggest there has been no important, if any, impact of varicella vaccination on overall medically attended herpes zoster rates. More in-depth analysis of Quebec data suggests some positive impact of varicella vaccination on incidence rates of medically attended herpes zoster cases in children (a decrease of 21% in children aged 0–9 years). Because the period of time since varicella vaccination programs implementation in Canadian provinces is relatively short (11–16 years), the longer term impact of these programs on herpes zoster burden at this point is unknown.

The large populations included in provincial databases used in eleven published Canadian studies and in our analysis (overall >30 millions) and their wide geographical distribution allow us to presume that the observed results are representative for Canada. Overall, this review shows relatively small variations in the burden of herpes zoster during the last 3-4 decades. In our opinion, the main factors influencing these variations and trends are the aging population, healthcare systems organization changes, and new available treatments. With an aging population and no efficient prevention in place, the burden of herpes zoster is expected to increase.

Herpes zoster incidence does not seem to follow geographic trends [20, 21], and the results of this systematic review support this observation. A recent systematic review of studies conducted worldwide shows that herpes zoster incidence varies but stays in a certain range. The lowest incidence rate (131 cases per 100,000 p.y.) was reported in an older study conducted in the United States between 1945 and 1959, and the highest was reported in a South Korean study (997 cases per 100,000 p.y.) between 2003 and 2007 [20]. An European review showed incidence rates between 200 and 457 cases per 100,000 p.y. [21]. The lowest and the highest of these incidence rates were reported in Iceland (1990–1995) and Belgium (1994–2003), respectively. Overall, Canadian incidence rates were in the same range with those reported in many other countries, including those reported in the United States [22]. The consistency of our results with those from other countries and geographical regions suggests our estimate of the burden of herpes zoster from the healthcare system perspective is valid and might be helpful when deciding about future preventive interventions.

Consistently with previous published data [23, 24], a Canadian study shows increased risk for herpes zoster in patients with common chronic diseases including diabetes and chronic obstructive pulmonary disease [19]. Furthermore, one recent study confirms the relatively low short-term-only effectiveness of the live attenuated vaccine. With such low effectiveness and an estimated vaccine uptake of 8% among persons aged 60 years and over [7], little impact on herpes zoster burden might be expected. The latter is at least partially supported by little variation in herpes zoster burden in the last 3-4 decades.

This review and analysis has some limitations. First, we limited our search to the peer-reviewed literature. As such, some data reported in the grey literature could have been missed. Second, the studies included and the Quebec analysis were based on data retrieved from existing administrative databases. This approach is subject to potential information biases, which may have impacted the results. In the Quebec study, as well as in the ten studies included in this systematic review, the herpes zoster diagnosis codes have not been validated. However, in the recent Canadian study using the CPCSSN data, the ICD-9 code 053 has been shown to have a sensitivity of 100% and a positive predictive value of 84% [19]. Some studies conducted in the United States found similar results for the ICD-9 code 053 with a sensitivity of 98% and a positive predictive value varying between 84% and 94% [5, 25–27]. These figures suggest that specific ICD codes may be reliable to estimate herpes zoster incidence. Third, a given proportion of herpes zoster cases without medical care seeking are not reported to databases. Thus, the obtained results reflect herpes zoster burden from the healthcare perspective only and do not allow to estimate the societal burden. Fourth, in order to verify for a potential double counting, we used two definitions for an incident case (i.e., first-time reported case during a calendar year and first-time reported case during the entire study period). Both definitions are arbitrary, and the second one does not include recurrent cases. However, the difference of 8.9% in cases captured with the first definition and the second definition is congruent with recently published results from a large study, which reported a recurrence of the disease in 6.4% of patients [28]. This suggests that our main analysis with the definition of an incident case as the first diagnosis during a calendar year estimates reasonably well both first-time and recurrent medically attended cases of herpes zoster.

## 5. Conclusion

The results of the reviewed studies and our analysis of Quebec provincial data indicate important variations in the reported overall incidence rates of medically attended herpes zoster cases in Canada. The trends in time are heterogeneous in studies in which hospitalisations and medical consultations were pooled together. The results are more consistent in studies which analysed the hospitalisations and medical consultations separately. The latter studies' results suggest a decrease in hospitalization rates and a slight increase in consultation rates. In our opinion, these trends are mainly explained by changes in the management of consulting cases, improved access to ambulatory medical services, and availability of antiviral medication. The results of this review and Quebec analysis consolidate the understanding of the herpes zoster burden in Canada and might be used as a tool in decision-making regarding preventive interventions including future cost-effective evaluations of potential immunization programs.

## Figures and Tables

**Table 1:** Canadian studies presenting herpes zoster incidence rates of outpatient visits and hospitalizations.

Author and year of publication	Periods; province; number of incident herpes zoster cases (*N*)	Cases identification	Incidence rates (per 100,000 person-years) (standardized unless stated otherwise)		
			Consultations (outpatient visits)	Hospitalizations	Outpatient visits + hospitalizations
Brisson et al. 2001 [11]	1979–1997; Manitoba; *N*: not documented	(i) Annual billing claims database; first claim with a herpes zoster ICD-9 code (ii) Hospital length of stay ≥ 1 day; hospitalisations with herpes zoster (HZ) ICD-9 code in one of the first three positions	*Crude rates* (i) All ages: increase from 258 to 348 during the study period (ii) ≥ 65 years: average rate of 812	*Crude rates* (i) ≥ 65 years: average rate of 86	Not documented

Russell et al. 2007 [15]	1986–2002; Alberta; *N*: not documented	(i) Provincial registration database (ii) Physician claims data system (iii) Hospital morbidity inpatient database (iv) First-time healthcare service utilisation for an HZ ICD-9/10 code in any position	Not documented	(i) All ages: decrease of hospitalized cases from 5% in 1993 to 3% in 2002	*Crude rates* (i) All ages: increasing rate from 1986 to 2002 (roughly from 280 to 440 according to the presented figure)

Edgar et al. 2007 [4]	1994–2003; British Columbia; (i) *N* of outpatient visits = 114,596 (ii) *N* of hospitalizations = 3,887	(i) Physician billing data; physician visit for an HZ ICD-9 code (ii) Hospitalisations for an HZ ICD-9/10 code regardless of its position (iii) Physician visits and hospitalizations ≥ 365 days apart were included	(i) All ages: increase from 324 to 366 (ii) ≥ 65 years: average crude rate of 703	(i) All ages: decrease from 12 to 8 (ii) ≥ 65 years: average crude rate of 51	Not documented

Brisson et al. 2008 [12]	2000–2003; Manitoba; *N*: not documented	(i) Hospitalisations for an HZ ICD code in the first position vs. any position	Not documented	*Crude rates among ≥ 65-year-olds* (i) HZ code in any position: average rate of 3 for 65–69 years and 30 for ≥80 years (ii) HZ code in the first position: average rate of 1 and 9 for 65–69 years and ≥ 80 years, respectively	Not documented

Tanuseputro et al. 2011 [17]	1992–2010; Ontario; (i) *N* of outpatient visits = 686,763 (ii) *N* of hospitalizations = 14,240	(i) First outpatient visit for an HZ ICD-9 code (ii) First hospitalization for an HZ ICD-9/10 code in any position (in a sensitivity analysis: hospitalization with HZ as primary diagnosis only)	(i) All ages: decrease from 525 to 479 (ii) ≥ 60 years: average crude rate of 740 during 2005–2009	(i) All ages: decrease from 9 to 4 (and from 5 to 2 in a sensitivity analysis with HZ as primary diagnosis)	(i) All ages: steady mean annual rate between 309 during 1992–1998 (prevaricella vaccine availability) and 303 during 2005–2009 (publicly available vaccine) Rate ratio (2005–2009 vs. 1992–1998) = 0.98 (0.82–1.14)
Russell et al. 2014 [14]	1994–2010; Alberta; *N* of outpatients and inpatients = 174,711	(i) Supplemental enhanced service event system (physician claims records) (ii) Alberta communicable disease reporting system (iii) Morbidity and ambulatory care reporting databases (hospital inpatients and hospital emergency department visits) (iv) First health service utilisation for an HZ ICD-9/10 code in any position (v) HZ codes ≥ 180 days after the first diagnosis classified as recurrent episodes	Not documented	(i) All ages: decrease of hospitalized cases from 5% during prelicensure of the varicella vaccine period (1994–1998) to 3% during the publicly available varicella vaccination period (2002–2010)	*Crude rates (recurrent episodes included)* (i) All ages: increase from 350 to 450

Antoniou et al. 2014 [18]	1997–2009; Ontario; *N* = 19,143	(i) Prescription drugs database (ii) Physician claims records database (iii) Hospitalisation admissions database (iv) Emergency department visits database (v) New diagnosis of HZ: first HZ ICD-9/10 code or first antiviral treatment for HZ	Not documented	Not documented	*Crude rates* (i) ≥ 66 years: average rate of 1,232 (1,171 for patients not receiving statin and 1,325 for statin users)

Marra et al. 2016 [10]	1997–2012; British Columbia; *N* = 238,295	(i) Medical services plan and hospital discharge database (ii) PharmaNet (outpatient prescription database) (iii) HZ ICD-9/10 code in any position without any evidence of HZ or postherpetic neuralgia in the previous 12 months (iv) Sensitivity analysis: HZ diagnosis with antiviral treatment received within 7 days	Not documented	(i) All ages: decrease of hospitalized cases from 4% during prelicensure of the varicella vaccine period (1997–1999) to 2% during the publicly funded vaccination period (2005–2012)	(i) All ages: increase from 316 to 449 (and from 162 to 300 in a sensitivity analysis with case definition as an HZ diagnosis with an antiviral treatment) Rate ratio (publicly funded varicella vaccination vs. prelicensure period) = 1.22 (1.20–1.24)
Friesen et al. 2016 [13]	1997–2013; Manitoba; *N*: not documented	(i) Physician claims database (ii) Hospital discharge database (iii) First HZ ICD-9/10 code in any position (iv) Multiple episodes included if a minimum of 2 years had elapsed since the first-time HZ and ≥ 180 days had elapsed since the last HZ diagnosis	Not documented	Not documented	(i) ≥ 20 years: steady rate at 470 from 1997-1998 to 2008-2009 and increase since 2009-2010 to reach 570 in 2013-2014

McDonald et al. 2017 [16]	2009–2015; Alberta; *N* of outpatients and inpatients = 49,243	(i) Physician claims database (ii) Morbidity and ambulatory care reporting database (iii) First HZ ICD-9/10 code in any position	Not documented	Not documented	*Crude rates* (i) ≥ 50 years: average rate of 903 (ii) Incidence rates increased from 581 in 50–54 years to 130 in ≥ 75 years

Queenan et al. 2017 [19]	2013–2015; Canadian Primary Care Sentinel Surveillance Network; *N* of outpatients = 3,281	(i) Surveillance system electronic database (ii) HZ ICD-9 code in any position	*Prevalence in ≥ 18 years* (i) One year: 0.32% (ii) By age: (1) 18–39 years: 0.12% (2) 40–64 years: 0.35% (3) ≥ 64 years: 0.67% (iii) By status: (1) No chronic disease: 0.21% (2) Diabetes: 0.69% (3) COPD: 0.69% (4) Any neoplasm: 1.07% (5) HIV/AIDS: 1.00%	Not documented	Not documented

Quebec analysis	1996–2015; Quebec; *N* of outpatients and inpatients = 543,290	(i) Physician claims records; first HZ ICD-9/10 code during a calendar year or during the entire study period (ii) Hospital discharge database; first HZ ICD-9/10 code as the main diagnosis or the first secondary diagnosis	Not documented	(i) All ages: decrease from 9 to 6 (ii) ≥ 65 years: decrease from 50 to 31	(i) All ages: decrease from 369 during 1996–2000 (prelicensure of varicella vaccine) to 350 during 2006–2015 (publicly funded varicella vaccination period) Rate ratio (2006–2015 vs. 1996–2000) = 0.95 (0.94–0.95) (ii) ≥ 65 years: average rate of 949 (steady over the study period)

## Data Availability

Only denominated data from administrative databases were available for the analysis.

## Abbreviations

RAMQ: Régie de l'Assurance Maladie du Québec
MED-ECHO: Maintenance et Exploitation des Données pour l'Étude de la Clientèle Hospitalière
ISQ: Institut de la statistique du Québec
p.y.: Person-years.
